# The Effect of a Brief Salivary α-Amylase Exposure During Chewing on Subsequent *in Vitro* Starch Digestion Curve Profiles

**DOI:** 10.3390/ijms11082780

**Published:** 2010-07-26

**Authors:** James W. Woolnough, Anthony R. Bird, John A. Monro, Charles S. Brennan

**Affiliations:** 1 Food Futures National Research Flagship and CSIRO Food and Nutritional Sciences, Adelaide, Australia; E-Mails: James.Woolnough@csiro.au (J.W.W.); Tony.Bird@csiro.au (A.R.B.); 2 New Zealand Institute for Plant & Food Research Limited, Palmerston North, New Zealand; 3 Institute of Food, Nutrition and Human Health, Massey University, Palmerston North, New Zealand; E-Mail: John.Monro@plantandfood.co.nz; 4 Department of Food & Tourism Management, Manchester Metropolitan University, Manchester, UK

**Keywords:** digestion, in vitro, saliva, starch

## Abstract

There is inconsistency between current *in vitro* digestion methods with regard to accommodation of a (salivary) α-amylase exposure during the oral phase. The effect of a salivary α-amylase pre-exposure on subsequent *in vitro* starch digestion curve profiles for various foods was investigated. Foods were chewed, expectorated and the boluses left to rest for 0–15 min. During pancreatic digestion, aliquots were taken and hydrolysis curves constructed for comparison against those of the same foods comminuted with a manually-operated chopper, hence spared exposure to saliva. Hydrolysate aliquots taken at T_0_ (time zero) of the digestion of chewed samples contained higher levels of glucose and dextrins compared with chopped samples. Pancreatin activity immediately overwhelmed differences in sugar released due to salivary amylase activity. Within 10 min no differences were detectable between hydrolysis curves for chewed and chopped foods. Salivary amylase pretreatment does not contribute to the robustness or relative accuracy of *in vitro* methods.

## 1. Introduction

*In vitro* carbohydrate digestion methods provide a time- and cost-efficient means for analysing the carbohydrate digestibility and hence likely glycemic properties of foods. *In vitro* digestions indicate how a given food is likely to behave *in vivo*, in terms of the rate and extent of sugar release from available carbohydrates, by simulating physiological processes occurring in the mouth, stomach and small intestine. There are many examples in the literature of *in vitro* technologies being used in various applications such as investigating the effect of food processing techniques on carbohydrate digestibility [1,2] and the digestive properties of foods fortified with ingredients thought to be beneficial to health [3,4], and explaining differences in blood glucose responses measured in human trials [5–7]. However, a comparison of any of the commonly used and referred to *in vitro* methods quickly reveals numerous methodological differences between protocols. Such differences include the method of sample comminution, whether a pepsin digest is included, the choice of amylolytic enzyme used and duration of simulated intestinal digestion, as well as the method used for stirring digests. It is likely that such differences in methodology influence the relative digestibility results a given *in vitro* method provides. The effect of varying simulated chewing techniques, for instance, on *in vitro* digestion curve profiles for various foods has already been demonstrated [8].

One aspect of methodology currently not consistent between *in vitro* methods is exposure to (salivary) α-amylase prior to simulated gastric phase digestion. *In vivo*, the chewing process features both a mechanical aspect, major disruption of food structure through combined action of the teeth and tongue, and a chemical/enzymic aspect, mixing of chewed foods with saliva containing the starch-hydrolysing enzyme α-amylase. The net effect of chewing is to reduce the particle size of food and create a suitably-sized, lubricated bolus safe for swallowing. Rather than attempt to simulate this process, a number of *in vitro* methods employ volunteers to chew and expectorate the test samples prior to *in vitro* digestion [9,10]. Volunteer chewing, however, is generally regarded as a highly subjective process that varies with individual. Considerable research effort has therefore been aimed at characterising the mechanical aspect of chewing in terms of the degree of food particle size reduction occurring [11,12] with the goal of producing via simulated chewing techniques, “as eaten” particle sizes prior to *in vitro* digestion.

Simulated chewing techniques such as mincing [5,6,13], chopping [8] and sieving [14] have been employed because they are easy to use and consistent in the particle size reduction they achieve. However, not all methods incorporate exposure to salivary α-amylase, inherent to chewing. Some methods using simulated chewing techniques include a brief α-amylase pre-exposure before progression to gastric digestion [15,16]. Hoebler *et al*. [17] for instance, mixed minced bread samples with specific amounts of pancreatic amylase consistent with amylase activity measured in saliva during the mastication of bread [18]. Just as it has been shown that the mode of sample comminution can affect relative *in vitro* estimates of digestibility [8], it is possible that inconsistencies in the degree of salivary exposure during oral phase treatment will also translate into conflicting relative *in vitro* results.

The present study aimed to quantify the amount of starch digested by salivary α-amylase during a typical chewing cycle, and determine its influence on, or the effect of omitting it from, the *in vitro* estimation of the glycemic impact of foods. *In vitro* digestion curves for chewed samples given different times of exposure to saliva are compared with those from saliva-free samples of similar particle size to assess the impact of salivary pre-exposure on subsequent *in vitro* digestion curve profiles.

## 2. Materials and Methods

### 2.1. Sample Preparation

Five test foods were bought from local markets: white bread, whole wheat grains, pasta, chick peas and potato. Foods were selected to span a range of glycemic index (GI) values. Where appropriate, foods were prepared by boiling in an excess of water: chick peas (after an overnight soak) and potatoes 40 min, whole wheat grains (after an overnight soak) 30 min and pasta sheets 10 min. After cooking, potatoes were peeled and chickpeas de-husked.

### 2.2. Simulated Chewing–No Exposure to Salivary Amylase

Following cooking, a subset of the test foods were artificially chewed using a manually-operated, domestic kitchen food chopper (*Zyliss*^®^). The device is operated by placing a portion of the food to be chopped in a small round plastic chamber. Above the chamber is a zigzag-shaped stainless steel blade that slices through the chamber, cutting the food, with each depression on the chopper handle. In previous work [8] we compared 20 chops with actual chewing, and found there to be no difference in *in vitro* carbohydrate digestion rates as influenced by particle size for potato, chick pea and bread. Pasta, however, required 47 chops and whole wheat grains 116 in order to resemble chewed samples in terms of equivalent particle size reduction.

### 2.3. Chewing – Exposure to Salivary Amylase for Designated Period

A series of pre-weighed 5.0 g samples of each of the test foods (2.5 g for bread based on the high glycaemic index of bread) were chewed by two volunteers with normal dentition until they reached the urge to swallow. The samples were then expectorated and left to sit as chewed boluses for 0, 5, 10 and 15 min. Prolonged bolus sitting periods were included to reflect the time taken *in vivo* to swallow, and for food to become fully mixed with gastric secretions, up to which time salivary α-amylase is likely still digesting starch. Between each sample, volunteers rinsed their mouths thoroughly with distilled water.

### 2.4. In Vitro Digestion

*In vitro* digestions involving simulated gastric and intestinal conditions [13] were carried out in 70 mL plastic biopsy pots inserted to their full depth in wells in an aluminium heating block preheated to 37 °C, on a 15-place magnetic stirrer. Duplicate 5.0 g samples (2.5 g in the case of bread) of test foods that had been chopped, and the entire chewed samples, were each placed into the pots and 30 mL of distilled water added, followed by 0.8 mL of 1 M aqueous HCl, the pH adjusted to 2.5 if necessary. Then 1 mL of a 10% pepsin (porcine, *Sigma*, P 7000; 800–2,500 U/mL) solution in 0.05 M HCl was added and the samples incubated at 37 °C for 30 min with slow constant stirring (130 rpm) to simulate gastric digestion conditions. After 30 min, the pH was adjusted to 6.0 with the addition of 2 mL of 1 M NaHCO_3_ and 5 mL of 0.1 M Na maleate buffer. A 100 μL dose of amyloglucosidase (A.niger, *Megazyme*, E-AMGDF; 3260 U/mL) was added to prevent end-product (maltose) inhibition of pancreatic amylase, followed immediately by 5 mL of a 2.5% pancreatin (porcine, *Sigma*, P7545; 8 × USP specifications) solution in 0.1 M Na maleate buffer, pH 6.0. The final digest volume was adjusted to 55 mL with distilled water. Simulated intestinal digestion proceeded at 37 °C for 120 min with slow constant mixing (130 rpm). Aliquots of 1.0 mL were withdrawn at 0 (T0; before adding pancreatin/amyloglucoidase), 10, 20, 40, 60 and 120 min and added to test tubes containing 4 mL absolute ethanol for later reducing sugar analysis by dinitrosalicyclic (DNS) colourimetry.

### 2.5. Reducing Sugar Determination Using DNS Colourimetry

The test tubes containing 1 mL digesta aliquots in 4 mL absolute ethanol were centrifuged at 2,500 rpm for 3 min. Aliquots of 0.05 mL were withdrawn from each tube and transferred into a fresh set of tubes, as were water blanks, reagent blanks and glucose standards. To each tube, 0.25 mL of a 1% invertase, 1% amyloglucosidase in 0.1 M Na acetate buffer pH 5.2 (‘enzyme solution A’ [ESA]) was added. Tubes were gently shaken and left to sit at room temperature for 15 min while any incompletely hydrolysed starch fragments were broken down to measurable glucose (secondary digest). Further 0.05 mL aliquots were taken from all T_0_ digesta samples (aliquots taken before pancreatin addition and therefore containing only free sugars as well as glucose and starch fragments released by salivary action), but to this set no ESA was added so only free sugars (present in the food before chewing) and glucose liberated by salivary action were present for subsequent measurement. DNS mixture (0.75 mL) containing 0.5 mg/mL glucose: 4 M NaOH: DNS reagent, mixed in the ratio 1:1:5, was added to all tubes, the tubes covered and boiled in a water bath for 15 min. Following boiling, samples, reagent blanks and glucose standards were diluted with 4 mL water before transferring to cuvettes and their absorbance (abs) read by colourimetry at 530 nm wavelength.

Total reducing sugar concentrations in each aliquot were calculated using the following formula and expressed as milligrams of reducing sugar per gram of food. The multiplication by 55 reflects the total volume (mL) of digest, 10 to reference the reading to the 10 mg/mL glucose standard and 5 to account for the dilution of the 1 mL digest aliquot into 4 mL absolute ethanol.

$$\frac{\left(\text{sample abs}-{\text{H}}_{2}\text{O blank abs}\right)}{\left(10\;\text{mg}/\text{mL standard abs}-{\text{H}}_{2}\text{O blank abs}\right)}\times 55\times 10\times 5\times 1/\text{original}\;\text{sample weight }(\text{g})$$

Free food reducing sugars were determined as the amount of reducing sugar measured in T_0_ aliquots of a given food after chopping (no saliva), and no secondary ESA digest. Glucose released by salivary action was determined as the total reducing sugar measured in T_0_ aliquots of a given food after chewing with no secondary ESA treatment, minus free food reducing sugars. Dextrins, or fragments of partly digested starch, released by salivary action, were determined as the reducing sugars measured following secondary ESA digestion of T_0_ aliquots from chewed samples, minus reducing sugar in the same samples spared the secondary ESA digestion. Values for the total starch (TS) content of each food were taken from the Australian Food Composition Tables 2006 [19]. The percentage of TS hydrolysed by salivary α-amylase to glucose and dextrins was calculated by converting the TS content (g/100 g) to absolute glucose content per 5 g of sample, then dividing the values for glucose released by salivary action or dextrins by the absolute glucose content, and multiplying by 100.

### 2.6. Statistics

All calculations and statistical analyses were performed using *Genstat* software. Mean values for reducing sugar concentration at each time point were calculated from the duplicate measures. A repeated measures analysis of variance (ANOVA) was carried out to compare pretreatment method with time, and least significant differences (LSDs) for the comparison of different treatments at given time points were calculated. A one-factor ANOVA was used to compare respective T_0_ (no ESA) measures of reducing sugar for each food, and a two-factor ANOVA was used to compare differences within a treatment between T_0_ (ESA) and T_0_ (no ESA) measures for a given food. All significant differences are reported at a significance level of 0.05.

## 3. Results and Discussion

The percentage of total starch hydrolysed by salivary amylase to glucose (no ESA treatment) in each chewed treatment category is shown in Table 1 whereas Table 2 reports the percentage of total starch hydrolysed to glucose plus dextrins (ESA treatment) in each chewed treatment category.

The results indicate that although some samples showed significant difference in starch hydrolysis from freshly chewed samples and chewed samples left for five minutes (for instance bread and potato) this was not common for all the samples tested (Tables 1 and 2). The effect of standing the chewed samples in expectorate for prolonged times (from +5–+15 min) did not have a significant effect on the levels of glucose (Table 1) or glucose and dextrins (Table 2).

**Figure 1:**
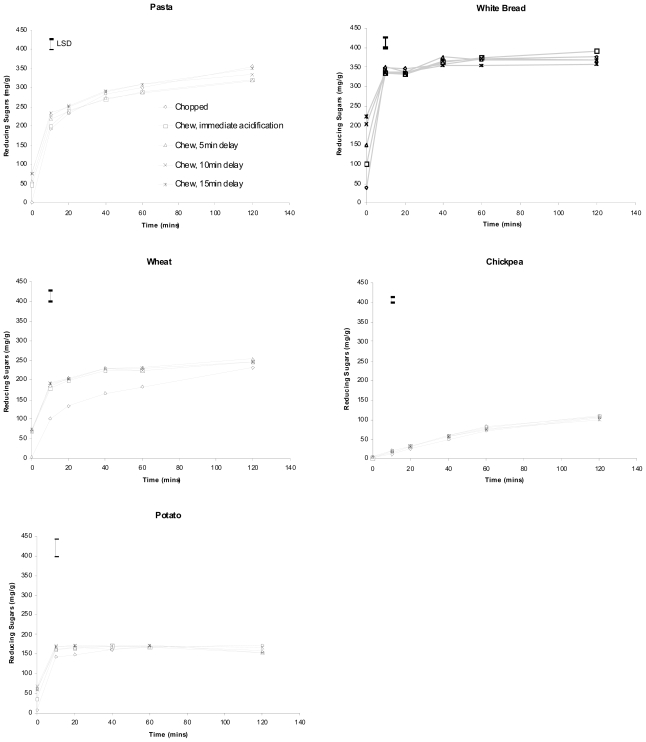
Effect of differences in the degree of salivary exposure during the oral phase on subsequent *in vitro* digestion curve profiles of different foods^a^.

**Figure 2:**
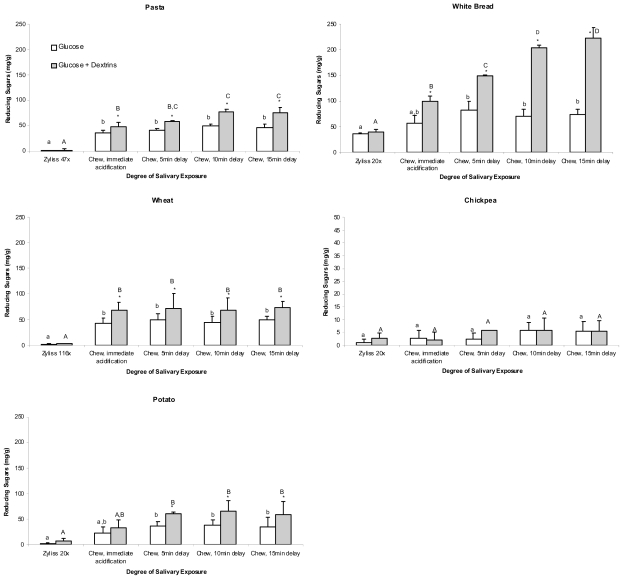
The amounts (mg/g) of glucose and dextrins released by salivary action during chewing of different foods^a^. ^a^ Reducing sugars measured in the chopped treatment category are the free sugars naturally occurring in the foods and so therefore may not exclusively consist of glucose. Different letters denote a significant difference between corresponding bars in different treatments. An asterisk denotes a significant difference between bars within a treatment. Error bars are standard error of the mean (n = 2). Error bars are least significant differences (LSD) between the treatments at each timepoint. Pasta LSD = 27.67. White bread LSD = 28.53. Wheat LSD = 28.97. Chickpea LSD = 14.79. Potato LSD = 44.18.

Hydrolysis curves for the five foods following chopping and chewing are shown in Figure 1. Concentrations of free sugars, glucose and dextrins measured in T_0_ aliquots of the five foods are shown in Figure 2.

Figures 1 and 2 illustrate that a significant increase in the amount of reducing sugars (glucose) in T_0_ (no ESA) samples from chewed pasta, bread, wheat and potato was measured compared with those of the corresponding chopped samples of these foods (*p* ≤ 0.017). However, no such significant increase at T_0_ was measured when chickpeas were chewed. Prolonged salivary exposure (5, 10 or 15 min) did not contribute to significantly greater amounts of glucose in any of the foods studied.

A secondary digest of T_0_ samples from chickpea using ESA did not cause a significant increase in reducing sugars measured regardless of whether the chickpea was chopped or chewed, and regardless of the duration of post-chewing salivary exposure. Chopped samples of the remaining foods also showed no significant increase in reducing sugars at T_0_ following a secondary ESA digest when compared with respective T_0_ (no ESA) samples. A secondary ESA digest of T_0_ samples from chewed pasta, bread, wheat and potato, however, did elicit significantly greater measures of reducing sugar when compared with their respective T_0_ (no ESA) samples (*p* ≤ 0.001). For pasta and white bread, this significant effect was further increased with prolongation of salivary exposure.

Any significant effect observed between chopped and chewed T_0_ samples was lost within 10 min of pancreatic *in vitro* digestion (20 min for pasta) as indicated by values for least significant difference (LSD). No interaction between pretreatment and digestion time was found for chickpeas and potato (Figures 1 and 2). Bread, however, showed differences in hydrolysis curve pattern both between chopped and chewed samples (*p* < 0.001) as well as among the chewed (*p* < 0.001). Wheat showed a significantly lower reducing sugar average when chopped (*p* < 0.001) and a different pattern between chopped and chewed (*p* < 0.001). Pasta showed differences in pattern between the chopped and chewed samples (*p* < 0.001).

During chewing and subsequent pre-gastric bolus resting, salivary α-amylase demonstrated varying capacity to digest starch from the test foods into at least short chains of partly digested starch (dextrins), and in some cases to glucose monosaccharide in a manner dependent on food type and duration of salivary exposure (Table 2 and Figure 2). A key finding was that including pre-exposure to salivary α-amylase by chewing did not affect the subsequent rate or extent of starch digestion from the foods *in vitro* as indicated by hydrolysis curve profiles, when compared with those of the saliva-free, chopped samples.

Simulated intestinal digestion rapidly obscured any differences in glucose released due to different durations of salivary digestion before initiation of the pancreatic digestion. This is important because *in vitro* estimates of a given food’s carbohydrate digestive properties, such as the starch fractions RDS, SDS, and RS, are commonly based on 20 min and 120 min periods of digestion [5,20], shown here to not be affected by even prolonged (15 min) salivary pre-exposure. The results show a point of robustness in *in vitro* methodology and indicate that for the sake of convenience it is not necessary to exactly replicate salivary α-amylase action occurring during normal chewing. Simple simulated chewing techniques may be used provided they achieve identical particle size reduction as chewing, and the transient influence of salivary α-amylase may be ignored. The slight differences in hydrolysis pattern observed with bread and pasta are not likely a result of salivary influence but of small dissimilarities in relative particle size distributions occurring between chopped and chewed samples as well as between different chewed samples of each test food.

Differences between chewed and chopped samples after 20 min of pancreatic digestion persisted in whole wheat (Figure 1) showing that it is necessary to carefully replicate chewed food structure for some food types. Wheat samples that had been chopped 116 times gave significantly lower measures of reducing sugars than chewed samples across the entire duration of *in vitro* digestion. Differences of this magnitude are not likely to have been caused by salivary action in chewed samples releasing sustained higher levels of glucose, but are more likely a result of differences in particle size affecting the overall rate and extent of starch digestion in wheat. In previous (unpublished) studies we compared the *in vitro* digestion rate of chewed samples with those prepared by varied repetitions (20–50) of chops. The rates of digestion of chickpea, white bread and potato were not sensitive to the number of chops, and digested at the same rate as chewed samples of those foods. Pasta and wheat, however, being more structurally robust foods, even after cooking, were sensitive to the number of chops, and extrapolation studies predicted that 47 chops (instead of the standard 20 for the softer foods) would adequately simulate chewing in terms of particle size reduction for pasta, and that wheat grains would require 116 chops. These predictions were accurate for pasta, but not for wheat, as shown in Figure 1, which shows the overall rate of *in vitro* digestion was greatly retarded due most likely to chopped wheat particles still being, on average, much larger than chewed wheat particles. This particular result for wheat highlights the importance of achieving “as eaten” particle sizes following simulated chewing, especially when structurally robust foods are being analysed. Chewing involves a combination of both cutting and crushing forces, whereas chopping only involves cutting. Thus despite the wheat being cut into quite small particles, it is likely many cells remain unruptured and hence resistant to digestion.

It was expected that exposure to salivary α-amylase in chewed samples would result in increased measures of glucose at T_0_ compared with chopped samples. While there was a trend for this to occur, a significant effect in the case of chewing and immediate acidification was only observed in pasta (the significant effect observed in wheat having to be discredited due to the failure of chopping to accurately replicate chewing for this food type). Prolonged pre-gastric exposure to salivary amylase (5 min) was required before a significant increase in glucose was measurable at T_0_ in white bread and potato. In none of the foods did pre-gastric exposure to salivary amylase of greater than 5 min duration result in higher levels of glucose at T_0_. It may be that the overall duration of exposure to saliva was the rate-limiting factor for glucose release and that further prolongation of exposure beyond 15 min would have resulted in higher levels of glucose being released in the foods studied. Equally likely, however, is the possibility that enzyme specificity of salivary α-amylase to just α,1–4 glycosidic bonds [21] limits further release of glucose from the foods regardless of whether exposure time is increased. This is perhaps demonstrated in the case of pasta and white bread, where increased exposure to saliva generated increased measures of partially-digested starch fragments and yet the amounts of glucose being measured over this time period remained the same.

The concentration of reducing sugars measured at T_0_ of pancreatic digestion in chopped samples, free of amylolytic enzyme – salivary amylase or pancreatin, and when ESA treatment is omitted – can be said to be the amount of free sugar occurring in that food. It follows, then, that any increase in sugars in corresponding T_0_ readings for chewed samples will have occurred as a result of starch being digested by salivary α-amylase during chewing. This increase in sugars (glucose) hence defines a portion of “extremely rapidly digestible starch” that is hydrolysed by salivary α-amylase during the brief chewing cycle and measurable as simple reducing sugars at T_0_. The partly digested fragments of starch, measured after a secondary ESA digestion, could also be included in this definition of extremely rapidly digestible starch, since they too are produced by salivary action. Certainly a portion of starch that is rapidly digested by saliva during chewing, and perhaps even in transit through the stomach, would contribute to the almost immediate glycemic response to food ingestion in fasted subjects [22,23], since this portion is already in the liquid phase of digesta upon arrival in the duodenum. Salivary amylase may continue to digest starch whilst in the stomach, as long as it is protected from the acid environment by being encapsulated in a bolus of food. It is this possibility that lends some physiological relevance to our leaving chewed boluses to sit for up to 15 min before progressing to the acidic gastric phase of *in vitro* digestion.

## 4. Conclusions

The activity of salivary α-amylase during chewing contributes to up to 43% of the total starch in foods being hydrolysed to simple sugars (glucose) and short chains of partly digested starch (dextrins). The degree of digestion depends on food type and duration of salivary exposure. Initiation of simulated intestinal digestion by adding pancreatin rapidly overwhelms any effect of saliva, such that the relative profiles of sugar released from foods during *in vitro* digestion do not differ significantly regardless of the degree of salivary pre-exposure. Therefore, in *in vitro* digestion methods where chewing is simulated, a salivary α-amylase enzyme step need not be included. The fraction of starch that is hydrolysed to glucose and dextrins by salivary action during the oral phase may be termed “extremely rapidly digestible starch,” and will contribute to the fraction responsible for the rapid onset of glycemic response following ingestion of food.

## Figures and Tables

**Table 1:** Percentage of total starch hydrolysed to glucose by salivary α-amylase. Percentages are rounded to the nearest whole number. Ch = chewed.

Food	Ch	Ch + 5 mins	Ch + 10 mins	Ch + 15 mins
**Chickpea**	1	1	4	3
**Bread**	4	10	7	8
**Pasta**	11	13	16	15
**Wheat**	17	19	17	19
**Potato**	15	25	27	24

**Table 2:** Percentage of total starch hydrolysed either completely to glucose or partially to dextrins by salivary α-amylase. Percentages are rounded to the nearest whole number. Ch = chewed.

Food	Ch	Ch + 5 mins	Ch + 10 mins	Ch + 15 mins
**Chickpea**	0	2	2	2
**Bread**	13	23	34	38
**Pasta**	15	28	25	24
**Wheat**	27	28	26	28
**Potato**	20	39	43	38

## References

[1] Niba LL Processing effects on susceptibility of starch to digestion in some dietary starch sources Int. J. Food Sci. Nutr 2003 54 97 109 12701241 10.1080/096374803/000042038

[2] Kingman SM Englyst HN The influence of food preparation methods on the *in-vitro* digestibility of starch in potatoes Food Chem 1994 49 181 186

[3] Brennan MA Merts M Monro J Woolnough JW Brennan CS Impact of guar gum and wheat bran on the physical and nutritional quality of extruded breakfast cereals Starch/Stärke 2008 60 248 256

[4] Tudorica CM Kuri V Brennan CS Nutritional and physicochemical characteristics of dietary fiber enriched pasta J. Agric. Food Chem 2002 50 347 356 11782206 10.1021/jf0106953

[5] Englyst KN Englyst HN Hudson GJ Cole TJ Cummings JH Rapidly available glucose in foods: an *in vitro* measurement that reflects the glycemic response Am. J. Clin. Nutr 1999 69 448 454 10075329 10.1093/ajcn/69.3.448

[6] Araya H Contreras P Alvina M Vera G Pak N A comparison between an *in vitro* method to determine carbohydrate digestion rate and the glycemic response in young men Eur. J. Clin. Nutr 2002 56 735 739 12122549 10.1038/sj.ejcn.1601386

[7] Jenkins DJ Wolever TM Thorne MJ Jenkins AL Wong GS Josse RG Csima A The relationship between glycemic response, digestibility, and factors influencing the dietary habits of diabetics Am. J. Clin. Nutr 1984 40 1175 1191 6507340 10.1093/ajcn/40.6.1175

[8] Woolnough JW Monro JA Brennan CS Bird AR Simulating human carbohydrate digestion *in vitro*: a review of methods and the need for standardisation Int. J. Food Sci. Tech 2008 43 2245 2256

[9] Akerberg AKE Liljeberg HGM Granfeldt YE Drews AW Bjorck IME An *in vitro* method, based on chewing, to predict resistant starch content in foods allows parallel determination of potentially available starch and dietary fiber J. Nutr 1998 128 651 660 9482777 10.1093/jn/128.3.651

[10] Muir JG Birkett A Brown I Jones G O’Dea K Food processing and maize variety affects amounts of starch escaping digestion in the small intestine Am. J. Clin. Nutr 1995 61 82 89 7825543 10.1093/ajcn/61.1.82

[11] Xu WL Lewis D Bronlund JE Morgenstern MP Mechanism, design and motion control of a linkage chewing device for food evaluation Mech. Mach. Theory 2008 43 376 389

[12] Hoebler C Devaux MF Karinthi A Belleville C Barry JL Particle size of solid food after human mastication and *in vitro* simulation of oral breakdown Int. J. Food Sci. Nutr 2000 51 353 366 11103300 10.1080/096374800426948

[13] Mishra S Monro J Hedderley D Effect of processing on slowly digestible starch and resistant starch in potato Starch 2008 60 500 507

[14] Brighenti F Casiraghi C Baggio C Resistant starch in the Italian diet Brit. J. Nutr 1998 80 333 341 9924275 10.1079/096582198388283

[15] Lebet V Arrigoni E Amado R Digestion procedure using mammalian enzymes to obtain substrates for *in vitro* fermentation studies Lebensm-Wiss Technol 1998 31 509 515

[16] Brighenti F Pellegrini N Casiraghi M Testolin G *In vitro* studies to predict physiological effects of dietary fibre Eur. J. Clin. Nutr 1995 49 S81 S88 8549566

[17] Hoebler C Lecannu G Belleville C Devaux MF Popineau Y Barry JL Development of an *in vitro* system simulating bucco-gastric digestion to assess the physical and chemical changes of food Int. J. Food Sci. Nutr 2002 53 389 402 12396464 10.1080/0963748021000044732

[18] Hoebler C Karinthi A Devaux MF Guillon F Gallant DJG Bouchet B Melegari C Barry J-L Physical and chemical transformations of cereal food during oral digestion in human subjects Brit. J. Nutr 1998 80 429 436 9924264 10.1017/s0007114598001494

[19] NUTTAB 2006 Australian food composition tables Food Standards Australia New Zealand Canberra, BC, ACT 2610, Australia 2006

[20] Englyst HN Kingman SM Cummings JH Classification and measurement of nutritionally important starch fractions Eur. J. Clin. Nutr 1992 46 S33 S50 1330528

[21] Kandra L Alpha-amylases of medical and industrial importance J. Mol. Struct.-Theochem 2003 666 487 498

[22] Brand-Miller JC Stockmann K Atkinson F Petocz P Denyer G Glycemic index, postprandial glycemia, and the shape of the curve in healthy subjects: analysis of a database of more than 1000 foods Am. J. Clin. Nutr 2009 89 97 105 19056599 10.3945/ajcn.2008.26354

[23] Aston LM Gambell JM Lee DM Bryant SP Jebb SA Determination of the glycaemic index of various staple carbohydrate-rich foods in the UK diet Eur. J. Clin. Nutr 2007 62 279 285 17426747 10.1038/sj.ejcn.1602723 PMC2699495

